# Identification of novel proteins in the *Dictyostelium discoideum* chemorepulsion pathway using REMI

**DOI:** 10.17912/micropub.biology.000557

**Published:** 2022-05-05

**Authors:** Sara A Kirolos, Shiri Procaccia, Kyra E Groover, Rheeturaag Das, Ramesh Rijal, Richard H Gomer

**Affiliations:** 1 Department of Biology, Texas A&M University; 2 Faculty of Biology, Technion - Israel Institute of Technology

## Abstract

Chemorepulsion, the biased migration of a cell away from a signal, is essential for many biological processes and the ability to manipulate chemorepulsion could lead to new therapeutics for a variety of diseases. However, little is known about eukaryotic cell chemorepulsion. Utilizing the model organism *Dictyostelium discoideum,* we previously identified an endogenous chemorepellent protein secreted by *D. discoideum* cells called AprA, and proteins involved in the AprA-induced chemorepulsion pathway including the G protein-coupled receptor GrlH, G beta and G protein alpha 8 protein subunits, protein kinase A, components of the mammalian target of rapamycin complex 2 (mTORC2), phospholipase A, PTEN and a PTEN-like phosphatase (CnrN), a retinoblastoma orthologue (RblA), extracellular signal-regulated kinase 1 (Erk1), p-21 activated protein kinase D (PakD), and the Ras proteins RasC and RasG. In this report, we used a genetic screen to identify 17 additional proteins involved in the AprA-induced chemorepulsion pathway *.*

**Figure 1. Identification of novel proteins involved in the D. discoideum chemorepulsion pathway.**
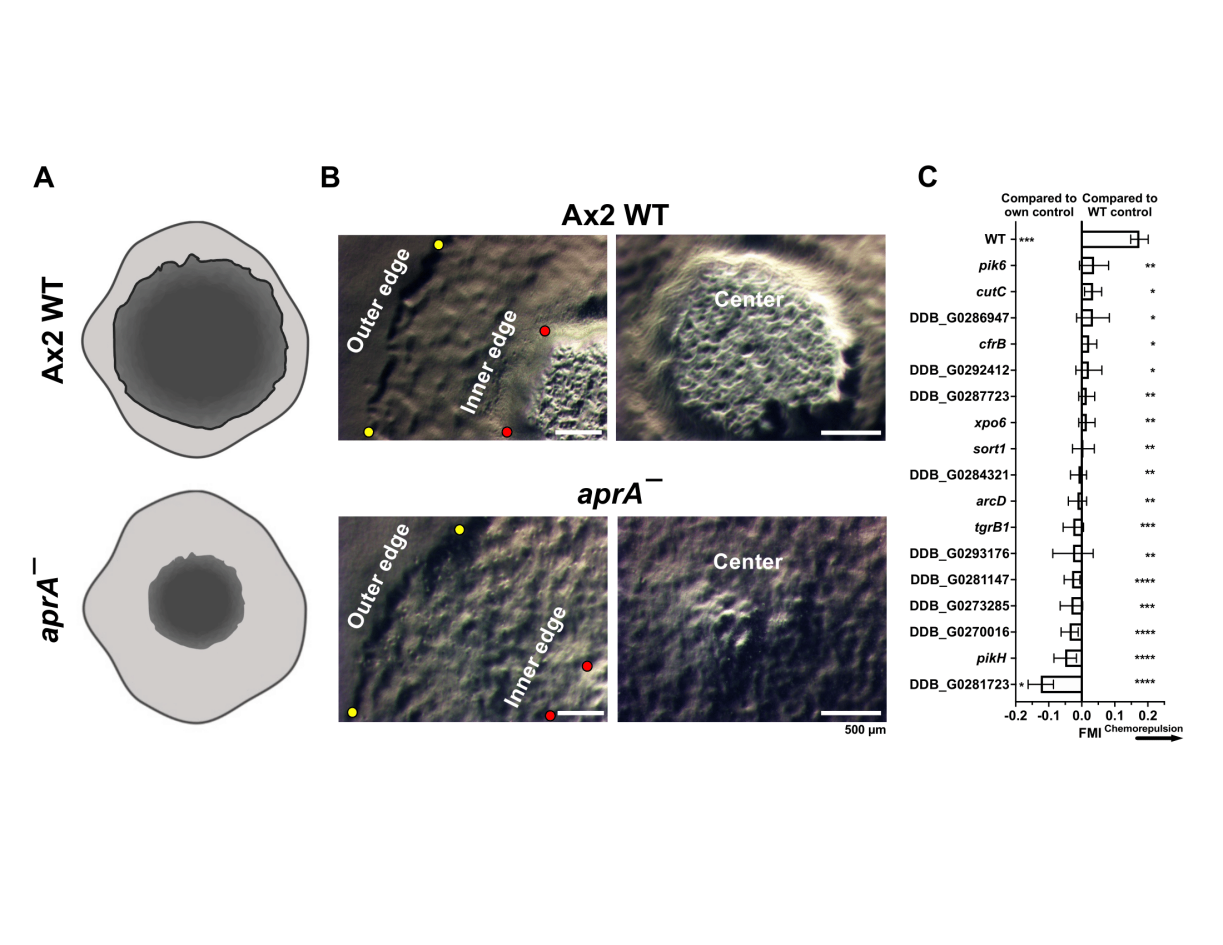
Restriction enzyme mediated insertional mutagenesis (REMI) was performed on parental Ax2 cells, and after selection for a successful insertion of a blasticidin-resistance cassette (and blasticidin resistance), transformants were spread on SM/5 plates with *E. coli* to generate plaques of clonal colonies. **(A)** A schematic of the primary screening of Ax2 WT and *aprA¯* colony phenotypes by colony edge morphology analysis. **(B)** Images of Ax2 WT and *aprA¯* colonies. Left column shows the outer edge (yellow dots) and inner edge (red dots) differences between the colonies, and the right column is representative images of the center of the colonies. Bars are 500 micrometers. **(C)** Well-separated individual cells of the indicated *aprA¯* -like mutants were imaged by videomicroscopy in growth medium (control) or in a recombinant AprA (rAprA) gradient in growth medium in Insall chambers. A positive forward migration index (FMI) indicates chemorepulsion from the AprA and a negative FMI indicates chemoattraction. Values are mean ± SEM of the averages of 3 independent experiments with at least 50 randomly chosen cells examined in each experiment. Inverse PCR was used to identify the gene disrupted by insertion of the REMI construct. On the right side of the graph, * indicates p < 0.05, ** p < 0.01, *** p < 0.001, and **** p < 0.0001 compared with Ax2 WT and on the left side of the graph, * indicates p < 0.05 and *** p < 0.001 comparing FMI in the presence and absence of a rAprA gradient (Unpaired t-tests, Welch’s correction).

## Description

*Dictyostelium discoideum* is a widely utilized model organism for elucidating chemotaxis, generally chemoattraction (Bozzaro 2013). In *Dictyostelium* as well as other systems, compared to chemoattraction, relatively little is known about chemorepulsion (Rijal et al. 2019; Herlihy et al. 2015; Herlihy et al. 2013; Herlihy et al. 2017). Growing *Dictyostelium* cells secrete a chemorepellent protein called AprA, and in colonies of *Dictyostelium* cells, the high extracellular concentrations of AprA in the colony and low concentrations outside the colony form a gradient that causes cells at the edge of the colony to move away from the colony, allowing cells to disperse and find new sources of food (Kirolos and Gomer 2022; Tang et al. 2018; Phillips and Gomer 2012).

During AprA-induced chemorepulsion, AprA binds to the G protein-coupled receptor GrlH initiating downstream signaling pathways that require for normal chemorepulsion the G beta and G alpha 8 protein subunits, protein kinase A, components of the mTOR2, phospholipase A, Erk1, PakD, PTEN, CnrN, RblA, and the Ras proteins RasC and RasG (Bakthavatsalam et al. 2009; Phillips and Gomer 2014; Rijal et al. 2019; Tang et al. 2018; Herlihy, Tang, and Gomer 2013).

To identify novel proteins in the AprA-induced chemorepulsion pathway, we utilized the restriction enzyme mediated integration (REMI) system (Kuspa and Loomis 1992). REMI was developed to generate random insertions into restriction sites of the *D. discoideum* genome, causing random mutations. The REMI insertion DNA contains a blasticidin resistance cassette, and cells that contain the integrated plasmid DNA can then be selected for by growth with blasticidin, and the transformants can then be screened for a desired phenotype (Adachi et al. 1994). Therefore, we utilized REMI to insert random mutations to generate cells that are insensitive to AprA mediated chemorepulsion. Our primary screen isolated cell colonies with an *aprA¯* phenotype, characterized by a colony that has a small center and a large gap between the inner edge and outer edge of the expanding colony compared to Ax2 WT colonies (Figure 1 A and B). Blasticidin-selected transformants were spread on SM/5 plates with *Escherichia coli* K12 ( *E. coli* K12 *)* lawns and single colonies were left to grow. As the colony from each single clone expanded, we selected those that exhibited an *aprA¯* phenotype (Figure 1 A and B). From a total of approximately 30,000 transformants, 4,000 individual clones exhibited the *aprA¯* phenotype. The 4,000 individual clones were cultured to a cell density of 1x10 ^6^ cells/ml with 10 µg/ml blasticidin and were re-plated on SM/5 plates with *E. coli* K12 lawns and single colonies were left to grow. Out of 4,000 clones, 650 clones showed both colony expansion on the SM/5 plates and exhibited an *aprA¯* phenotype.

We then wanted to determine which REMI clones did not have a biased movement away from an AprA gradient, indicating that the disrupted gene encodes a protein required for the AprA chemorepulsion pathway. Ax2 WT and the *aprA¯* phenotype REMI mutants were exposed to a gradient of buffer (control) or 300 ng/ml of AprA in an Insall chamber (Figure 1C, Rijal et al. 2019; Kirolos and Gomer 2022). We previously found that the AprA concentrations used in this assay do not affect cell speed or persistence in the direction of movement (Rijal et al. 2019). For all of the mutants examined in this report, compared to WT, there was no significant difference in speed or persistence in buffer or an AprA gradient, indicating that the observed phenotypes are not due to a general motility defect. The movement of cells toward or away from the AprA source in the chamber was measured as the forward migration index (FMI), with a positive FMI indicating chemorepulsion. From the 650 REMI clones selected in the primary screening, 37 REMI clones were randomly selected and tested for insensitivity to an AprA gradient as a secondary screening. 26 out of the 37 REMI clones were insensitive to the AprA gradient compared to the control, suggesting that the REMI insertion, or another mutation, disrupted the AprA-induced chemorepulsion pathway. Genomic DNA from the 26 AprA-insensitive REMI clones was extracted and inverse PCR was performed to amplify the region of the disrupted gene adjacent to the REMI DNA. Amplified sequences from 17 of these REMI clones were obtained and sequenced (Figure 1C). For unknown reasons, we were not able to obtain inverse PCR products from the remaining 9 REMI clones.

The proteins identified as possibly being required for the AprA chemorepulsion pathway are: the PIP kinase Pik6; the copper transporter CutC, a predicted protein encoded by DDB_G0286947; I-TASSER (Yang et al. 2015) suggests that this has structural similarity to AnkB, which anchors membrane proteins to the cytoskeleton, the cell surface glycoprotein CfrB, DDB_G0292412 described on dictybase as similar to *S. cerevisiae* and *S. pombe* TRK1 and TRK2 potassium transporters, the nuclear exportin Xpo6, the intracellular protein sorting protein Sort1, the actin related protein 2/3 complex subunit 4 ArcD, the cell-cell adhesion and recognition protein TgrB1, a predicted protein encoded by DDB_G0293176; I-TASSER suggests that this has structural similarity to the mechanical forces sensing protein NompC, a predicted protein encoded by DDB_G0273285; I-TASSER suggests that this has structural similarity to the nuclear pore complex component Nup205, a predicted protein encoded by DDB_G0270016; I-TASSER suggests that this has structural similarity to the short-chain dehydrogenase SDR, and the PI3 kinase PikH, which is not involved in chemoattraction (Takeda et al. 2007). Four additional proteins in the Figure 1C list were uncharacterized proteins in dictybase that showed no predicted structural similarity to a known protein by I-TASSER.

Together, this data expands our understanding of eukaryotic chemorepulsion mechanisms, which play a crucial role in biological processes including development, morphogenesis and immune response. Since the observed phenotypes may be due to mutations not at the REMI insertion site, homologous recombination-mediated replacement of a large section of the coding region, or CRISPR/Cas9 disruption, and then rescue by expression of the corresponding cDNA, will be needed to conclusively prove that these candidates are indeed needed for chemorepulsion.

## Methods

*REMI assays*

Ax2 WT cells were grown to 5 x10 ^6^ cells/ml in HL5 (HLG0102, Formedium, Hunstanton, England) in shaking culture (Brock and Gomer 1999; Kirolos and Gomer 2022; Rijal et al. 2019). REMI was performed as previously described (Adachi et al. 1994; Kuspa 2006) using plasmid pBSR1 (Shaulsky, Escalante, and Loomis 1996) linearized with the restriction enzyme BamHI (#R0136S, New England BioLabs Inc., Ipswich, MA). Once the cells were selected for using 10 µg/ml blasticidin (#B-800-100, GoldBio, St. Louis, MO), the selected cells were grown to 1x10 ^6^ cells/ml and then spread at ~ 50 cells/ 10 cm diameter petri plate on SM/5 plates with *E. coli* K12, and single colonies were screened for the *aprA¯* phenotype described above. Colonies that had an *aprA¯* phenotype were picked and grown to 6-8 x10 ^6^ cells/ml in HL5 with 10 µg/ml blasticidin in shaking culture.

G *enomic DNA extraction and inverse PCR assays*

Genomic DNA was extracted from the growing colonies that showed abnormal chemorepulsion using Quick-DNA Miniprep Plus kits (#D4068, Zymo research, Irvine, CA). The extracted genomic DNA was digested using AluI restriction enzyme (#R0137L, NEB, Ipswich, MA) overnight at 35°C. The samples were then cleaned and concentrated using DNA Clean and Concentrator-5 kits (#D4013, Zymo Research). The samples were then ligated using T4 DNA ligase (#M0202S, NEB) overnight at 16°C. Inverse PCR to identify the REMI DNA insertion site was done following (Keim, Williams, and Harwood 2004). Inverse PCR was performed on the ligated samples with primers (see reagents section for sequences) that anneal to the cassette of the inserted plasmid (mentioned in the above section). The amplified samples were then run on 0.7% agarose gels (#0710-100G, VWR, Solon, OH). The amplified sequences were then gel purified using GeneJET gel extraction kits (#K0691, ThermoScientific, Waltham, MA). The samples were then sent for sequencing to identify the disrupted genes.

*Chemorepulsion assays*

Chemorepulsion assays on the *aprA¯* phenotype REMI clones were performed as a secondary screening as described in (Kirolos et al. 2021; Rijal et al. 2019).

## Reagents

**Gene list**

**Table:** 

**Gene nomenclature**	**Gene ID**
***pik6***	**DDB_G0275635**
***cutC***	**DDB_G0287539**
** DDB_G0286947 (I-Tasser: AnkB) **	**DDB_G0286947**
***cfrB***	**DDB_G0290259**
**DDB_G0292412 (I-Tasser: Trk1)**	**DDB_G0292412**
**DDB_G0287723**	**DDB_G0287723**
***xpo6***	**DDB_G0293076**
***sort1***	**DDB_G0290081**
**DDB_G0284321**	**DDB_G0284321**
***arcD***	**DDB_G0269102**
***tgrB1***	**DDB_G0280689**
**DDB_G0293176 (I-Tasser: NompC)**	**DDB_G0293176**
**DDB_G02811467**	**DDB_G0281147**
**DDB_G0273285 (I-Tasser: Nup205)**	**DDB_G0273285**
**DDB_G0270016 (I-Tasser: SDR)**	**DDB_G0270016**
***pikH***	**DDB_G0291093**
**DDB_G0281723**	**DDB_G0281723**

**Reagents**

**Table:** 

**Reagent**	**Category Number**	**Source**
HL5	HLG0102	Formedium
Blasticidin	B-800-100	GoldBio
Quick-DNA Miniprep Plus Kit	D4068	Zymo research
AluI	R0137L	NEB
DNA Clean and Concentrator-5 kit	D4013	Zymo research
T4 DNA ligase	M0202S	NEB
Agarose	0710-100G	VWR
GeneJET Gel Extraction Kit	K0691	ThermoScientific
BamHI	R0136S	New England BioLabs
Plasmid	pBSR1	Dictybase
Primers	Forward: 5’-TGTCGTTAGAACGCGGCTAC-3’Reverse: 5’-CGTCGATATGGTGCACTCTC-3’	EtonBiosciences
